# Performance evaluation of reverse osmosis technology for selected antibiotics removal from synthetic pharmaceutical wastewater

**DOI:** 10.1186/1735-2746-9-19

**Published:** 2012-12-10

**Authors:** Mitra Gholami, Roya Mirzaei, Roshanak Rezaei Kalantary, Ahmad Sabzali, Fateme Gatei

**Affiliations:** 1 Depatment of Environmental Engineering, School of Public Health, Center for Water Quality Research, Tehran University of Medical Sciences, Tehran, Iran; 2 Department of Environmental Health Engineering, School of Public Health, Isfahan University of Medical Sciences, Isfahan, Iran

**Keywords:** Amoxicillin, Ampicillin, Reverse osmosis (RO), Membrane technology, Pharmaceutical wastewater

## Abstract

This study addresses the possibility for low pressure reverse osmosis membrane (RE 2521, CSM) process to serve as an alternative to remove selected antibiotics (ampicillin and amoxicillin) from synthetic wastewater by changing operating conditions such as pH = 3, 6.5 and 10; Pressure = 9, 11 and13 (bar); antibiotic concentration = 10, 255 and 500(mg/L), and temperature = 20, 30 and 40°C. The experiment was designed based on Box-benken, which is a Response Surface methodology design (RSM), using Design Expert software. The concentration of antibiotics was measured by applying a UV-spectrophotometer (Cecil), at the wavelength of 254 nm. Results showed a range of rejection percentage from 73.52% to 99.36% and 75.1% to 98.8%, for amoxicillin and ampicillin, respectively. Considering the solute rejections and the membrane porosity show that the prevailing rejection mechanism of the examined antibiotics by the membrane was the size exclusion effect. The permeate flux for both of the antibiotics was 12–18.73 L/m^2^.h. Although the permeate flux and antibiotic rejection are influenced by operating pressure, pH, and temperature individually, the interaction between operating parameters did not have noticeable effects. According to the results obtained in this study, the application of RO membrane is recommended for the selected antibiotics to be removed to a considerable degree (up to 95%).

## Introduction

The effluents of Pharmaceutical industries are characterized by high organic matter contents, toxicity, deep color, and high salt contents. Among all the pharmaceutical compounds that have environmental concern, antibiotics have an important role due to their high consumption rates in both veterinary and human medicine. Development of antibiotic resistant bacteria is the worst problem that may be created by the presence of antibiotics at low concentrations in the environment [1]. Antibiotics are persistent and bioaccumulative contaminants and biologically activecompounds which have been developed to have an effect on organisms; hence, they have the potential to negatively affect either aquatic or tellurian ecosystems, even in low concentrations in the range of (μg–ng) per liter. In addition, antibiotics can also cause antibacterial resistance in microorganisms and be responsible for several allergenic responses [2-4].

Massive production of antibiotics started during World War II and so far, these compounds have been widely used in order to prevent and treat infectious diseases [4-7]. Although antibiotics have been used in large scale in the last fifty years, it was only in recent years that its occurrence in the environment became a subject of scientific and public relevance [4,8,9]. Over the past few years, antibiotics have also been considered as emerging pollutants due to their continuous input and persistence in the aquatic ecosystems even at low concentrations. Residues of antibiotics are present in a diversity of environmental matrices, like surface and groundwater, hospital and WWTPs effluents, soils and sediments [4,10-12]. Several antibiotics such as ampicillin, erythromycin, sulphamethoxazole, tetracycline and penicilloyl groups are mainly released into the environment by excretion (about 30–90%), reaching the wastewater treatment plants (WWTPs) where they are not completely removed, and contaminating natural waterways [9,13-15].

Amoxicillin and ampicillin are broad-spectrum b-lactam antibiotics that are semi-synthetic penicillin obtaining their antimicrobial properties from the presence of a beta-lactam ring [1].

They are commonly employed against infections caused by bacteria for human prescription medicine and as therapeutic agents due to their broad spectrum against bacteria [14]. Results of toxicology studies have revealed that some antibiotics such as amoxicillin are suspected to have direct toxicity to certain aquatic organisms such as algae [16,17]. It is reported that photosynthesis mechanism of algae *Synechocystis sp* is inhibitated by the toxic effects of this compound [18]. Furthermore, this compound accumulates within single organism, (i.e. pathogenic bacteria) and increases its resistance leading to higher dosage needed or even its incapability to treat conventional diseases [14,19]. Amoxicillin has also been known to be hardly degradable, remaining as active compound within urine and feces [14,20]. In addition, its continued discharge through industrial route finally leads to irreversible change on wildlife and human beings [14,19].

Based on The very limited efficiency of conventional treatment plants such as sand filtration, chemical coagulation/flocculation and chlorination, in removing various organic micropollutants (OMPs) such as antibiotics, advanced technologies such as ozonation, advanced oxidation processes (AOPs) and activated carbon are considered to be more efficient in eliminating polar pharmaceuticals. In addition pressure-driven membrane processes, particularly nanofiltration (NF) and reverse osmosis (RO) have also been gaining attention in the past decade and their application in drinking water treatment has been the focus of many researchers [21-23].

Regardless of the obvious advantages of using an RO membrane, which has been mostly used for desalination due to its ability to achieve high particulate rejection levels, it is suggested as an additional step in the sewage treatment process and it can be served as an absolute barrier to improve OMP removal. Several research groups have mentioned RO as the most promising and efficient technique for OMP removal [23-26].

The main contribution of this paper is to investigate the performance of a pilot scale RO membrane in rejection of selected antibiotics (ampicillin and amoxicillin) from a synthetic wastewater.

## Materials and methods

**Figure 1:**
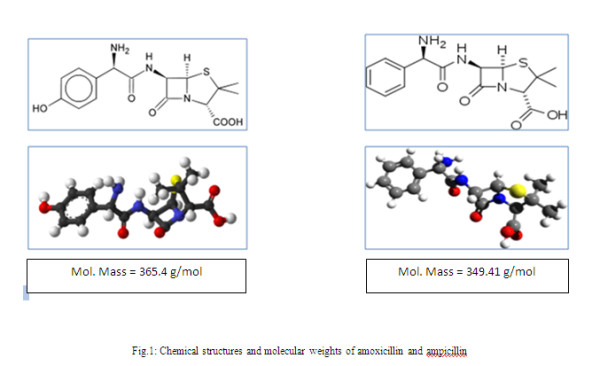
Chemical structures and molecular weights of amoxicillin and ampicillin.

In this study, two types of antibiotics- ampicillin and amoxicillin- which are the most produced with the highest rate consumed by Iranian pharmaceutical industry were considered. The chemical structure and molecular weights of amoxicillin and Ampicillin are shown in Figure 1.

The experiments were conducted using a cross flow spiral wound (RE2521) reverse osmosis membrane, supplied by CSM, a polyamide thin film composite membrane for operation at pH between 2 and 13 bar and temperature up to 40°C. The membrane characteristics are summarized in Table 1.

**Table 1:** Membrane characteristics

**Membrane type**	
Type	TFC(thin film composite)
Material	PA (polyamide)
Charge	Negative
Element	
Configuration	Spiral-wound, tapping
Size	Diameter: 2.5 inch; Length: 21 inch
Effective Area	1.1 m^2^ (12 ft^2^)

Chemicals were at least reagent grade and the highest purity commercially available. Both antibiotics were purchased from KOSAR pharmaceutical company in Iran. In order to prepare calibration curve, standard ampicillin and amoxicillin were supplied from Zigfride company, Germany. pH was adjusted with 1 mol/L NAOH and sulfuric acid. All solutions were prepared with tap water. Determination of antibiotic residues was conducted by spectrophotometric method, previously reported [22,27-30].

### Experimental setup

**Figure 2:**
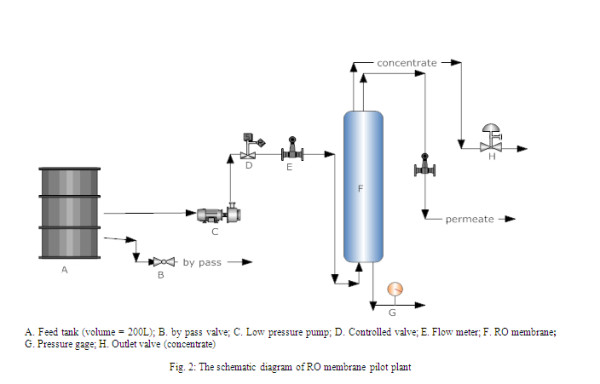
The schematic diagram of RO membrane pilot plant.

A pilot scale reverse-osmosis unit, schematically represented in Figure 2, was designed to evaluate the performance of the membrane. The membrane system unit was mainly comprised of a membrane module, pressure pump and a feed tank. Wastewater was pumped into the feed tank by means of a centrifugal high pressure pump, passing through the RO membrane.

The operating pressure over the membrane was varied from 9 to 13 bars, and the influent flow rate was changed from 0 to 8 L/min. Pressure and flow rates were set by manual valves.

The temperature was set at room temperature (20°C). The system was designed based on the hydraulic regime as the bench mark; therefore, there was no permeating or concentrate recycling. Experiments were carried out at three levels of concentrations for each antibiotic. After obtaining the optimum concentration, three different pressures at 20°C were studied. Also, the effect of pH and temperature on antibiotics removal efficiency was investigated. pH was adjusted by using sulfuric acid with normality of (1, 3) and sodium hydroxide with normality of (1, 3 and 6). The range of levels and variables are summarized in Table 2.

**Table 2:** Levels of experiments and range of operating parameters

**Variables Level**	**Pressure (bar)**	**concentration (mg/L)**	**pH**	**Temperature(°C)**
1	9	10	3	20
2	11	255	6.5	30
3	13	500	10	40

In all steps, samples were collected for analysis over a period of 2 hours [31].

### Sample analysis

The Box-Benken statistical design based on response surface method (RSM) was applied to determine runs of samples and data analysis. Factorial analysis was carried out to determine the significant factors and interactions between the factors that can be affected by the performance of RO membrane in terms of permeate flux and the antibiotic rejection. Through designing the experiments based on Box-benken, the investigation of the several factors affecting the efficiency of the system can be made possible. Simultaneous variation of the factors, rather than one at a time, can increase the efficiency of the experiment in terms of time and cost. Moreover, interactions between the factors can be easily studied in this way. Samples of permeate and raw wastewater were collected during experiments. Analytical approaches were based on the Standard Methods [32].

The concentration of the antibiotics was measured by means of a UV-spectrophotometer (CE-7400, Cecil) at the wavelength of 254 nm and pH was measured by HACH pH meter (HQ40d). Retention factor (R) of each compound and water flux was calculated by equations (1) and (2), respectively:

(1) $$R=\left[1-\frac{C_{P}}{C_{F}}\right]\times 100$$

Where R is the retention factor (%), Cp is the concentration in the permeate (mg/L) and C_F_ is the concentration in the raw solution (mg/L) [33,34]

(2) $$J_{W}=\frac{Q_{P}}{A}$$

Where J_w_ is the permeate flux (L/m^2^.h), Q_P_ is the permeate flow per hour and A is active surface area of membrane (m^2^) [35,36]. The relationship between permeate flux and operating pressure is based on equation (3) which was originally formulated by [37] as:

(3) $$J=A\left(P-\Delta \pi \right)$$

Where J, A, P, and p are the permeate flux, the water permeation constant, the applied pressure and the osmotic pressure, respectively. Osmotic pressure can be predicted by using the Van’t Hoff equation (4) as follows [38].

(4) $$\pi =V_{i}C_{i}\mathrm{RT}$$

Where V_i_, C_i_, R, and T are the number of ions formed if the solute dissociates, the molar concentration of solute, the gas constant, and the absolute temperature, respectively.

## Results

The results of performance reverse osmosis membrane for removal of selected antibiotics (ampicilin and amoxicillin) samples are shown in Tables 3 and 4. As can be seen, the variations of rejection and permeate flux are greatly influenced by parameters such as pH and operating pressure. The results were analyzed through factorial analysis, based on Design Expert Software.

**Table 3:** Experimental results of amoxicillin (with molecular weight of 365.4 g/mol) rejection by RO membrane conducted in different conditions and variables (all of runs are in block1)

**Std**	**Run**	**Factor 1 A:pressure (Bar)**	**Factor 2 B:concentration (mg/L)**	**Factor 3: C:pH**	**Factor 4 D:temprature (°C)**	**Response 1 rejection (%)**	**Response 2 permeate flux (L/m**^**2**^**.h)**
23	1	11	10	6.5	40	84.24	14.73
22	2	11	500	6.5	20	87.85	14.7
6	3	11	255	10	20	90.94	15.1
13	4	11	10	3	30	80.23	15.8
17	5	9	255	3	30	73.52	12.9
7	6	11	255	3	40	80.09	16.36
12	7	13	255	6.5	40	95.37	18.34
19	8	9	255	10	30	77.84	12.7
21	9	11	10	6.5	20	82.37	15.27
25	10	11	255	6.5	30	82.62	16.2
11	11	9	255	6.5	40	76.31	13.17
9	12	9	255	6.5	20	74.93	12.54
18	13	13	255	3	30	87.34	18.37
15	14	11	10	10	30	91.45	15.81
1	15	9	10	6.5	30	78.55	13.2
24	16	11	500	6.5	40	89.21	14.18
8	17	11	255	10	40	92.12	16
20	18	13	255	10	30	99.36	17.9
26	19	11	255	6.5	30	83.1	16.7
5	20	11	255	3	20	79.43	15.7
16	21	11	500	10	30	90.12	14.45
14	22	11	500	3	30	78.77	14.62
27	23	11	255	6.5	30	84.12	14.7
4	24	13	500	6.5	30	94.21	17.93
3	25	9	500	6.5	30	75.76	12.54
2	26	13	10	6.5	30	98.23	18.54
10	27	13	255	6.5	20	93.25	17.67

**Table 4:** Experimental results of ampicillin (with molecular weight of 349.41g/mol) rejection by RO membrane conducted in different conditions and variables (all of runs are in block1)

**Std**	**Run**	**Factor 1 A: pressure (Bar)**	**Factor 2 B: concentration(mg/L)**	**Factor 3 C:pH**	**Factor 4 D:temprature(°C)**	**Response1 rejection (%)**	**Response 2 permeate flux(L/m**^**2**^**.h)**
22	1	11	500	6.5	20	90.57	14.63
23	2	11	10	6.5	40	91.13	16.27
20	3	13	255	10	30	98.57	17.26
19	4	9	255	10	30	80.02	12
18	5	13	255	3	30	94.61	18.47
21	6	11	10	6.5	20	90.75	15.73
7	7	11	255	3	40	83.96	15.95
10	8	13	255	6.5	20	96.52	18.12
13	9	11	10	3	30	93.91	15.81
27	10	11	255	6.5	30	89.31	15.27
24	11	11	500	6.5	40	90.91	15.8
1	12	9	10	6.5	30	79.1	13.63
8	13	11	255	10	40	93.79	16.1
12	14	13	255	6.5	40	97.22	18.63
11	15	9	255	6.5	40	78.71	13.32
16	16	11	500	10	30	93.12	16.9
5	17	11	255	3	20	82.12	15.5
14	18	11	500	3	30	83.11	15.42
2	19	13	10	6.5	30	98.8	18.73
15	20	11	10	10	30	93.91	15.81
25	21	11	255	6.5	30	89.13	15.1
26	22	11	255	6.5	30	90.24	15.8
3	23	9	500	6.5	30	76.37	12.54
9	24	9	255	6.5	20	77.31	12
6	25	11	255	10	20	93.47	14.9
17	26	9	255	3	30	75.1	13.1
4	27	13	500	6.5	30	96.97	18.54

From the results in Tables 3 and 4, the rejection and permeate flux by RO membrane varied between 73.52% to 99.36% and 12.7 to 18.5 (L/m^2^.h) for amoxicillin and 75.1% to 98.8% and 12 to 18.73 (L/m^2^.h) for ampicillin, respectively.

As can be seen in Table 3, the effect of trans-membrane operating pressure on selected antibiotics’ rejection was much more than that of pH (76% to 96% for pressure increasing from 9 to 13 bar and 80% to 90% for pH of 3 to 10). It can also be seen that the effect of concentration and temperature on the rejection was by no means noticeable. The analysis of variance (ANOVA) for rejection and permeate flux of both antibiotics are shown in Tables 5 and 6, respectively. These results indicate the significance of the main operating parameters and their interaction effects based on p-value (at P < 0.05 level of significance).

**Table 5:** ANOVA for amoxicillin rejection and interaction effects of parameters

**Response 1 rejection**						
ANOVA for Response Surface 2FI Model						
Analysis of variance table for amoxicillin						
	Sum of		Mean	F	p-value	
Source	Squares	df	Square	Value	Prob > F	
Model	1370.63	10	137.063	28.9064	<0.0001	significant
A-pressure	1023.98	1	1023.98	215.955	<0.0001	
B-concentration	0.06021	1	0.06021	0.0127	0.9117	
C-pH	325	1	325	68.5419	<0.0001	
D-temperature	6.12041	1	6.12041	1.29078	0.2726	
AB	0.37823	1	0.37823	0.07977	0.7812	
*AC*	14.8225	1	14.8225	3.12603	0.0961	
AD	0.1369	1	0.1369	0.02887	0.8672	
BC	0.00423	1	0.00423	0.00089	0.9766	
BD	0.06503	1	0.06503	1.37E-02	0.9082	
CD	0.0676	1	0.0676	0.01426	0.9064	
Residual	75.8661	16	4.74163			
Lack of Fit	74.6925	14	5.33518	9.09199	0.1034	not significant
Pure Error	1.1736	2	0.5868			
Core Total	1446.5	26				

**Table 6:** ANOVA for ampicillin rejection and interaction effects of parameters

**Response 1 rejection%**						
ANOVA for Response Surface 2FI Model						
Analysis of variance table for ampicillin						
	Sum of		Mean	F	p-value	
Source	Squares	df	Square	Value	Prob > F	
Model	1307.76	10	130.776	20.2892	< 0.0001	significant
A-pressure	1122.88	1	1122.88	174.209	< 0.0001	
B-concentration	22.8252	1	22.8252	3.54122	0.0782	
C-pH	133.8	1	133.8	20.7585	0.0003	
D-temprature	2.0667	1	2.0667	0.32064	0.5791	
AB	0.2025	1	0.2025	0.03142	0.8615	
AC	0.2304	1	0.2304	0.03575	0.8524	
AD	0.1225	1	0.1225	0.01901	0.8921	
BC	25.05	1	25.05	3.88639	0.0662	
BD	0.0004	1	0.0004	6.21E-05	0.9938	
CD	0.5776	1	0.5776	0.08961	0.7685	
Residual	103.129	16	6.44558			
Lack of Fit	102.42	14	7.31568	20.6134	0.0472	significant
Pure Error	0.7098	2	0.3549			
Cor Total	1410.89	26				

The permeate flux decreased slightly as concentration was increased from 10 to 500 mg/L. According to ANOVA results for both of antibiotics’ permeate flux show that operating pressure and concentration have significant effect on permeate flux, based on P<0.0001 for pressure and P<0.04 for concentration. It can be concluded that pressure has a significant influence (more than 90 percent). The effect of pH and temperature on permeate flux were negligible.

**Figure 3:**
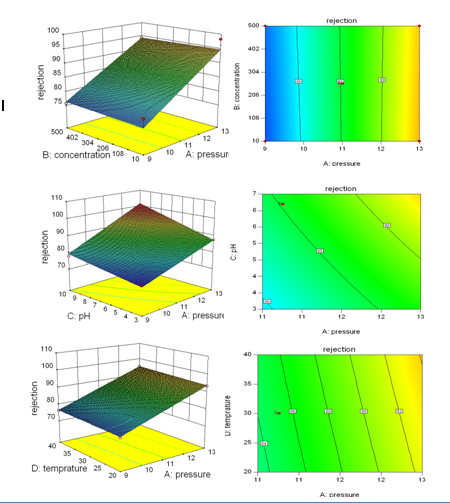
Three-dimensional surface plots (left column) and two dimensional contour plots (right column) showing the response surface function effects of the interactions between: A: pressure and concentration, B: pressure and pH, C: pressure and temperature on amoxicillin rejection.

**Figure 4:**
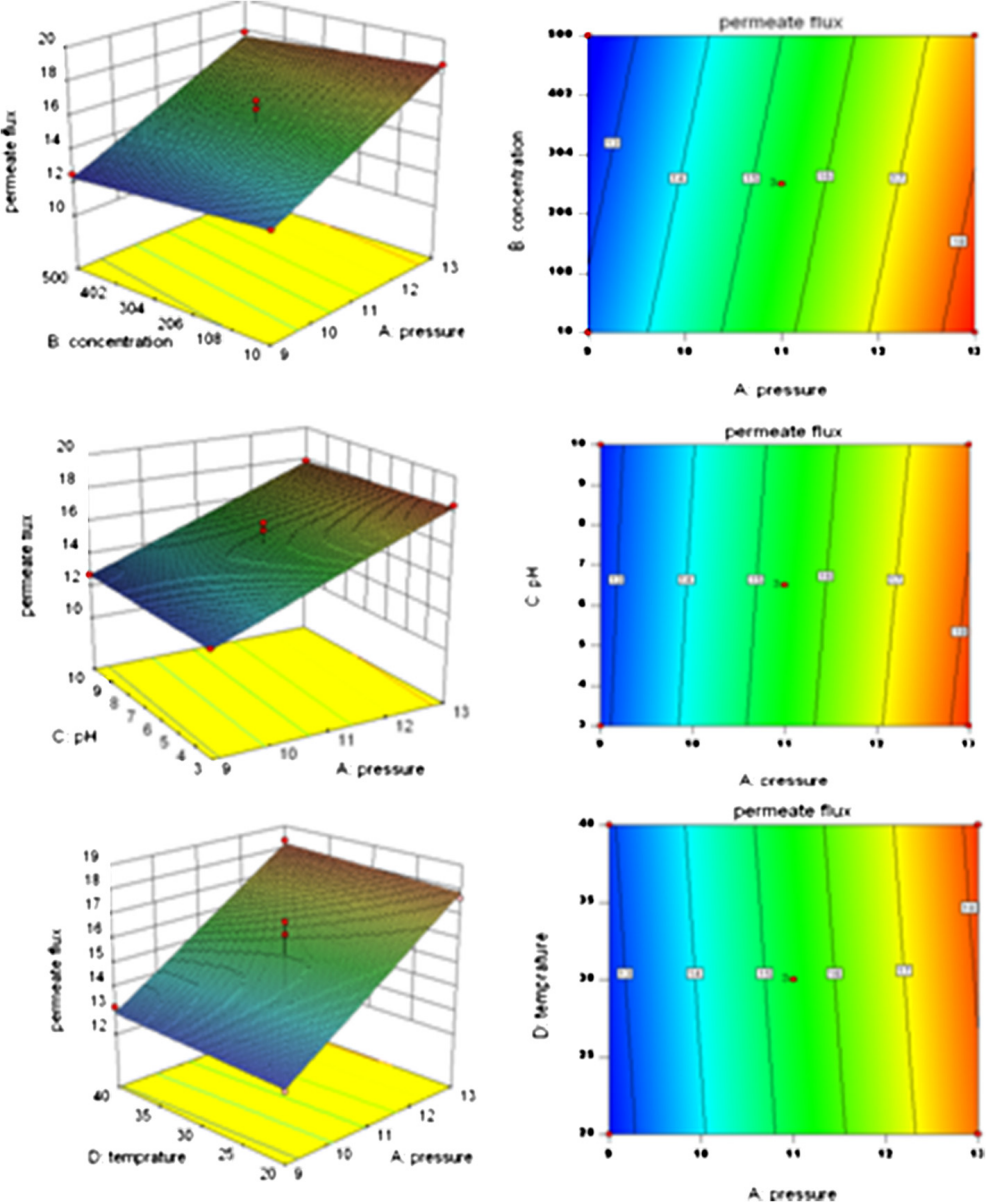
Three-dimensional surface plots (left column) and two dimensional contour plots (right column) showing the response surface function effects of the interactions between: A: pressure and concentration, B: pressure and pH, C: pressure and temperature on amoxicillin permeate flux.

The interactions among the parameters on the amoxicillin rejection and permeate flux in three-dimensional surface plots and two dimensional contour plots are shown in Figures 3 and 4.

## Discussion

Based on the results, removal efficiency increased with molecular weights of the antibiotics compounds. The best rejection was obtained for amoxicillin (99.36%), which has a higher molecular weight than ampicillin (365.4 g/mol). There is a consensus that nonionazable organic solutes with molecular weights between 200 and 400 g/mol are efficiently rejected by RO/NF membranes [39,40].

The results showed that an increase in pressure from 9 to 13 bar would lead the flux to increase as well, which was due to the solution-diffusion model. Also, the condensed membrane increases the static resistance and then more solutes are rejected [41]. On the other hand, flux under the experimental conditions is also a function of salt retention, as the retained ions accumulate in the boundary layer of the membrane where the concentration polarization effect imposes an osmotic pressure and reduces the effective driving force across the membrane [42]. By increasing feed pressure, the driving force will increase and the overcome membrane resistance [33,38,43,44]. In this study, increasing the pressure from 9 to 13 bar led to an increase in the permeate flux and rejection. Higher flux values were obtained at 13 bar for applied amoxicillin and ampicillin (18.54 L/m^2^.h); (Tables 3 and 4 for amoxicillin and ampicillin, respectively).

With respect to the experimental conditions used in this study, the P-value (< 0.001) showed that the pressure and pH have significant effects on the rejection of amoxicillin and ampicillin individually, but all interaction effects among the operating parameters in this study were insignificant for selected antibiotic rejection. As presented in Tables ( 5 and 6), P-values for all interactions were higher than 0.05.

Retention of organic pollutants in membrane separation processes depends on the characteristics of both membrane and the pollutants [45]. In addition, most of the papers reviewed by Bellona et al. have shown that the transport of uncharged organic compounds through reverse osmosis (RO) membrane is controlled mainly by the sieving mechanism [46]. However, the rejection of the uncharged organic by RO/NF membranes is often affected by physio-chemical properties of the system, and in the case of ionized organics, the charge exclusion plays a significant role in the rejection process [38,39]. The sieving mechanism of solute rejection is dependent on the relation between the size of solute molecules and the size of the membrane pores. An RO membrane has a very small molecular weight cut-off (MWCO) and it can retain a large fraction of low molecular weight compounds (e.g. amino acids or sugars). As pointed out by kimura et al, negatively charged compounds would be significantly rejected by NF/RO membranes due to the electrostatic repulsion between the compounds and membranes, even compounds with a small molecular weight (e.g. 110) and a rather loose membrane (i.e. NF) [38,47]. Therefore, molecular weight is one of the most important factors in antibiotic removal by RO membrane.

According to the obtained results, the degree to which the antibiotics were rejected increased as pH incresed. The phenomenon can be explained through the charged membrane and the charged solute which leads to a Donnan potential. Zeta potential of the membrane had a negative charge as the absolute zeta potential value decreased towards acidic pH values. This charge variation as a function of pH is due to the dissociation of membrane functional groups such as carboxylic and amide, and adsorption of hydroxide ion. All those effects were influenced by the pH of solution [48]. With respect to the chemical structure of antibiotics, acidic pH leads to the production of negative charged ions of antibiotics. The charged membrane attracts opposite charged ions to achieve equilibrium. At the same time, the membrane will repel the same charged ions by an electrostatic force. In addition, the opposite charged ions will also be rejected due to electorneutrality in the solution. Because of these interactions, the water can pass through the membrane. This mechanism enhances the rejection of antibiotics due to the charges of pH, causing the membrane to be charged [38].

Although the increase in the feed concentration had no effect on antibiotic rejection, permeate flux decreased slightly. It is known that when concentration increases, osmosis pressure will increase as well, decreasing the effective operating pressure. At the same time, the viscosity of solution will increase, leading the flux to decrease [41].

Based on Figures 3 and 4, the distribution of contours suggested that all of the parameters were quite independent of each and that the interactions between all of the parameters (pressure, concentration, pH, and temperature) were insignificant to the antibiotics rejection and permeates flux. Since the results obtained from experiments carried out on ampicillin were the same as those conducted on amoxicillin, repetitive results were omitted.

## Competing interests

The authors declare that they have no competing interests.

## Authors’ contributions

Mitra Gholami, Roya Mirzaei, Roshanak Rezaei Kalantary, Ahmad Sabzali and Fateme Gatei carried out the article with the title of: Performance evaluation of reverse osmosis technology for selected antibiotics removal from synthetic pharmaceutical wastewater, participated in the sequence alignment and drafted the manuscript. All authors read and approved the final manuscript.
